# Sex-specific associations of infants’ gut microbiome with arsenic exposure in a US population

**DOI:** 10.1038/s41598-018-30581-9

**Published:** 2018-08-22

**Authors:** Anne G. Hoen, Juliette C. Madan, Zhigang Li, Modupe Coker, Sara N. Lundgren, Hilary G. Morrison, Thomas Palys, Brian P. Jackson, Mitchell L. Sogin, Kathryn L. Cottingham, Margaret R. Karagas

**Affiliations:** 1 Department of Epidemiology, The Geisel School of Medicine at Dartmouth, Hanover, New Hampshire USA; 2 Children’s Environmental Health & Disease Prevention Research Center at Dartmouth, Hanover, New Hampshire USA; 3 Department of Biomedical Data Science, The Geisel School of Medicine at Dartmouth, Hanover, New Hampshire USA; 4 Division of Neonatology, Department of Pediatrics, Children’s Hospital at Dartmouth, Lebanon, New Hampshire USA; 5 Josephine Bay Paul Center, Marine Biological Laboratory, Woods Hole, Massachusetts, USA; 6 Department of Earth Sciences, Dartmouth College, Hanover, New Hampshire USA; 7 Department of Biological Sciences, Dartmouth College, Hanover, New Hampshire USA; 8 Present Address: Department of Biostatistics, University of Florida, Gainesville, Florida USA

## Abstract

Arsenic is a ubiquitous environmental toxicant with antimicrobial properties that can be found in food and drinking water. The influence of arsenic exposure on the composition of the human microbiome in US populations remains unknown, particularly during the vulnerable infant period. We investigated the relationship between arsenic exposure and gut microbiome composition in 204 infants prospectively followed as part of the New Hampshire Birth Cohort Study. Infant urine was analyzed for total arsenic concentration using inductively coupled plasma mass spectrometry. Stool microbiome composition was determined using sequencing of the bacterial 16S rRNA gene. Infant urinary arsenic related to gut microbiome composition at 6 weeks of life (p = 0.05, adjusted for infant feeding type and urine specific gravity). Eight genera, six within the phylum Firmicutes, were enriched with higher arsenic exposure. Fifteen genera were negatively associated with urinary arsenic concentration, including *Bacteroides* and *Bifidobacterium*. Upon stratification by both sex and feeding method, we found detectable associations among formula-fed males (p = 0.008), but not other groups (p > 0.05 for formula-fed females and for breastfed males and females). Our findings from a US population indicate that even moderate arsenic exposure may have meaningful, sex-specific effects on the gut microbiome during a critical window of infant development.

## Introduction

Arsenic is a ubiquitous environmental toxicant found in food and water worldwide^1–3^, and is present almost exclusively as inorganic arsenic in water supplies^4^, and as inorganic or organic species including metabolites of inorganic arsenic in dietary staples such as rice^5^. Environmental contaminants undergo biotransformation by gut bacteria. Biotransformation likely occurs with arsenic^6^ and both absorption and toxicity of arsenic are highly dependent on its speciation^4,7^. A human *in vitro* gut microbiome model suggests that bacteria may methylate inorganic arsenic into highly toxic species, such as monomethylarsonous acid, which absorbs into the blood stream^8^. Thus, the relationship between arsenic and the human microbiome is bidirectional – direct by its antimicrobial effects on gut microbes, affecting gut permeability and immunity, and indirect by increasing the concentrations of microbial metabolites of arsenic capable of affecting human health^1,4,6,8–10^.

Fetuses, infants and young children are especially vulnerable to the effects of contaminants such as arsenic (reviewed in^11^). Early life also is a critical window for establishment of the gut microbiome, and, concomitantly, priming of the immune system^12–21^. While the infant gut microbiome may develop partially in fetal life, colonization chiefly occurs during birth, early feeding and beyond^22–25^. Immune maturation and programming involve interaction with gut microbes, and alteration in early life colonization patterns has been linked to lifelong disease risk, including allergy, asthma, and obesity^24,26–33^.

Human arsenic exposure is a consequence of consumption of water and food containing arsenic. Infant formula powder and drinking water have been identified as important sources of arsenic exposure in infants^34,35^. US drinking water has varying levels of arsenic contamination and concentrations exceeding Environmental Protection Agency limits are particularly problematic in rural communities where private, unregulated wells commonly provide drinking water^13^.

Arsenic exposure is associated with effects on human health mediated through altered immunity, including infection, cancer, diabetes, cardiovascular disease, and in young children, impaired neurodevelopment^12,36–45^. Accumulating evidence indicate these effects occur as a consequence of arsenic exposure levels lower than previously appreciated. Investigations by our group of levels of arsenic exposure common in the US, beginning in fetal life through well water and maternal diet, identified potential health impacts that included dose-related trends in risk of infection and wheezing, as well as fetal growth restriction^12,13,42,46^. Immune profiling from this cohort has identified T cell decreases in cord blood^18^, increased placental gene expression of proinflammatory marker IL1B and developmental gene Gli3^48^, and DNA methylation changes in cord blood and placenta^20,48^.

In our comprehensive, molecular epidemiology study of over 1,500 pregnant women and subsequently, their offspring, we are investigating the effects of arsenic on pregnant women, their fetuses and infants. We are conducting the study in two regions of New Hampshire, including an area we previously determined has arsenic concentrations in well water that exceed 10 *μ*g/L, the Environmental Protection Agency maximum contaminant level for drinking water. Here, we hypothesized that infant arsenic exposure is associated with perturbations to the infant gut microbiome at 6 weeks of age and that these differences would be sex-dependent and most pronounced in formula-fed infants. Clarifying and quantifying human exposure and ultimate health impacts, including those that may be mediated by shifts in the composition of the microbiome, can lead to policy changes for protection of human health, especially in our most vulnerable populations.

## Results

As of June 17, 2016, 1,572 pregnant women had been enrolled in the NHBCS, and 1,544 NHBCS infants had reached 6 weeks of age. Inclusion criteria for the current study required the availability of infant urine and stool samples collected at 6 weeks of age and information on infant diet during the first 6 weeks of life. We began collecting 6-week stool samples on August 15, 2012, and of the enrolled infants, 205 met inclusion criteria for the current study; of these, one sample was removed due to sequencing failure, resulting in a final sample size of 204.

Selected subject characteristics are shown in Table 1. The mean urinary arsenic concentration among 204 infants aged 6 weeks was 0.6 *μ*g/L with a median of 0.4 *μ*g/L and range of below the limit of detection (<0.05 *μ*g/L) to 4.8 *μ*g/L. Three infants had urinary arsenic levels below the limit of detection. As we have demonstrated previously in this cohort, infant urinary arsenic levels were lowest among those who were exclusively breast fed^34^. Among the subjects included in the current study, infants who were exclusively breast fed had a mean urinary arsenic level of 0.5 *μ*g/L compared with a mean of 0.9 *μ*g/L for those fed both breast milk and formula and 1.2 *μ*g/L for those who were fed only formula.

**Table 1:** Selected characteristics of participants overall and by sex*.

Variable	All subjects (N = 204)	Males (N = 118)	Females (N = 81)
	Mean (Range) or N(%)	Mean (Range) or N(%)	Mean (Range) or N(%)
Gestational age, wk	39.3 (30.0–43.4)	39.3 (32.6–43.4)	39.6 (34.4–42.1)
Delivery mode			
Vaginal	140 (69)	82 (69)	58 (72)
Cesarean	55 (27)	34 (29)	17 (21)
Not reported	9 (4)	2 (2)	6 (7)
Infant birth weight, g**	3474 (1910–4689)	3496 (1910 4565)	3434 (2296 4689)
Feeding at 6 wk			
Exclusively breastfed	146 (72)	85 (72)	58 (72)
Combination fed	49 (24)	27 (23)	20 (25)
Exclusively formula-fed	9 (4)	6 (5)	3 (4)
Urinary arsenic concentration at 6 wk, ***μ***g/L	0.63 (<LOD – 4.8)	0.60 (<LOD – 3.9)	0.67 (<LOD – 4.8)
Home tap water arsenic concentration, ***μ***g/L	1.5 (<LOD – 57.0)	1.8 (<LOD – 57.0)	1.3 (<LOD – 23.1)

LOD: limit of detection (0.05 ***μ***g/L); *sex not reported for N = 5; **birth weight missing for n = 7 subjects.

Sequencing of the V4-V5 regions of the bacterial 16 S rDNA present in DNA extracted from infant stool samples collected at the same time yielded a total of 19,099,004 (mean: 93,623, range: 10,818–630,397) bacterial DNA reads that passed quality filtering procedures (details in^49^).

### Relationship between urinary arsenic concentration and infant stool microbiome composition at 6 weeks of age

**Figure 1:**
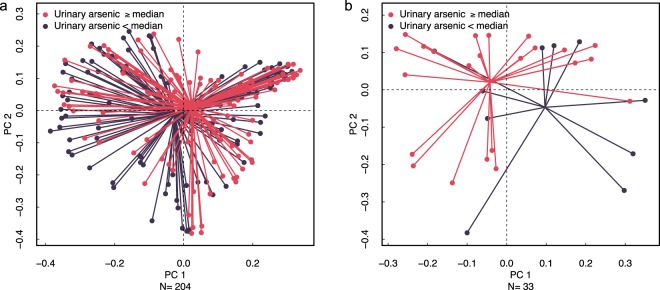
Principal coordinate plot of generalized UniFrac distance matrix comparing microbiome community profiles according to urinary arsenic concentration. (**a**) All N = 204 infants in the study; (**b**) N = 33 formula-fed males. Corresponding plots for other groups are presented in the supplementary information (Fig. S1). Statistical testing was performed using a continuous measure of arsenic exposure, the natural log of urinary arsenic concentration; however, for visualization purposes, here subjects were divided at the median urinary arsenic concentration of 0.36 *μ*g/L.

**Figure 2:**
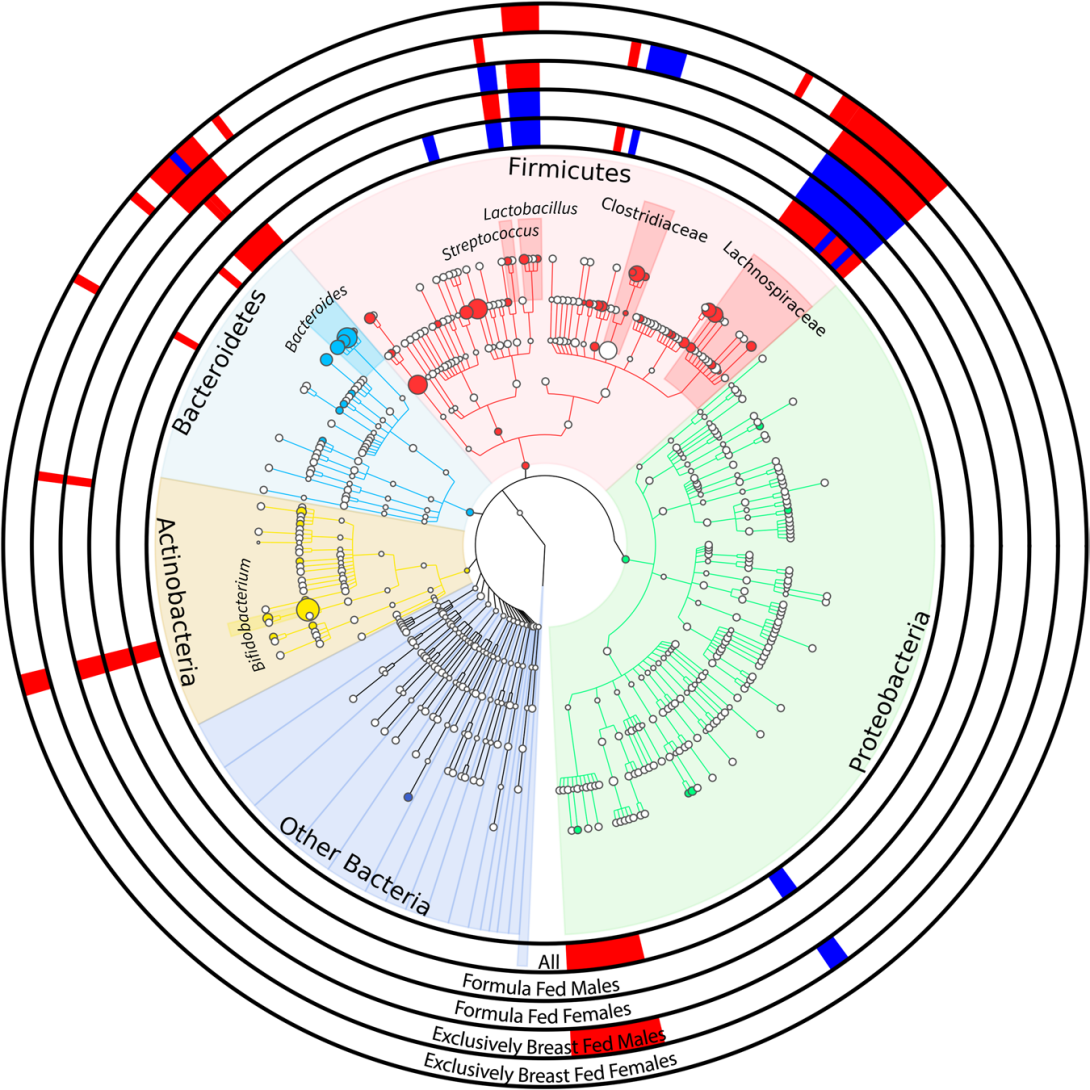
Cladogram showing bacterial OTUs with differential relative abundances based on arsenic levels. Color-shaded areas of the cladogram delineate the most abundant phyla in the infant gut microbiota, with the size of corresponding nodes proportional to the abundance level of the clades. Several OTUs were significantly associated with arsenic levels; bars in the outer rings identified individual OTUs that were positively (blue bars) and negatively (red bars) correlated with urine arsenic concentrations. Rings correspond to all infants in the study (N = 204), male formula-fed infants only (N = 33), female formula-fed infants only (N = 23), male exclusively breastfed infants only (N = 85) and female exclusively breastfed infants only (N = 58).

Among the 204 infants in our study, natural log-transformed urinary arsenic concentration was associated with infant stool microbiome adjusted for infant diet and urine specific gravity: pseudo *F* statistic: 1.85, p = 0.05; adjusted for urine specific gravity only: pseudo *F* statistic: 1.88, p = 0.05; Fig. 1a). We identified several associations between natural log-transformed infant urinary arsenic concentration and the relative abundance of individual bacterial taxa (Supplementary Table S1 and Fig. 2). Adjusting models for feeding status and urine specific gravity, we identified 8 OTUs with positive associations with natural log-transformed infant urinary arsenic concentrations at six weeks of age, including a strong positive association between natural log-transformed infant urinary arsenic and the relative abundance of bacteria in the genus *Ruminococcus*. We also identified 14 OTUs that were negatively associated with natural log-transformed infant urinary arsenic concentration, among them, OTUs assigned to the family Clostridiaceae and to the genera *Bacteroides* and *Bifidobacterium*. Overall, positively associated OTUs were largely from the phylum Firmicutes, while the majority of negatively associated OTUs were from the phyla Firmicutes or Bacteroidetes. Different OTUs representing bacteria of the genera *Lactobacillus* and *Dorea* were found to be both positively and negatively associated with natural log-transformed urinary arsenic concentration.

### Sex-specific relationships between urinary arsenic concentration and infant stool microbiome composition at 6 weeks of age in formula-fed infants

We stratified study subjects by sex to identify sex-specific effects given that sex-specific perturbations to gut microbiome community structure by arsenic exposure through drinking water have been observed in mice^50^. In addition, our previous research in the NHBCS indicated clear associations between the infant gut microbiome at 6 weeks of age and infant feeding and we also previously reported in this cohort that breast milk fed infants have lower urinary arsenic than formula-fed infants and is not a likely source of dietary arsenic^15,34^. Therefore, to address the potential for confounding or modification of the relationship between arsenic exposure and the infant microbiome by feeding method, we also stratified based on infant feeding, analyzing infants who did and did not receive formula separately. Thus, we analyzed four groups: formula-fed males, formula-fed females, exclusively breastfed males and exclusively breastfed females.

We found strong associations between natural log-transformed urinary arsenic concentration and infant stool microbiome composition among exclusively or partially formula-fed male infants (n = 33, adjusted for formula exclusivity vs breast milk with formula supplementation and urine specific gravity: pseudo *F* statistic: 2.30, p = 0.008; adjusted for urine specific gravity only: pseudo *F* statistic: 1.87, p = 0.03; Fig. 1b). No such association was observed in formula-fed females (n = 23, adjusted for formula exclusivity vs breastfed with formula supplementation and urine specific gravity: pseudo *F* statistic: 0.54, p = 0.93; adjusted for urine specific gravity only: pseudo *F* statistic: 0.56, p = 0.92; Fig. S1A); exclusively breastfed males (n = 85, adjusted for urine specific gravity: pseudo *F* statistic: 0.58, p = 0.83; Fig. S1B); or exclusively breastfed females (n = 58, adjusted for urine specific gravity: pseudo *F* statistic: 1.14, p = 0.39; Fig. S1C).

**Figure 3:**
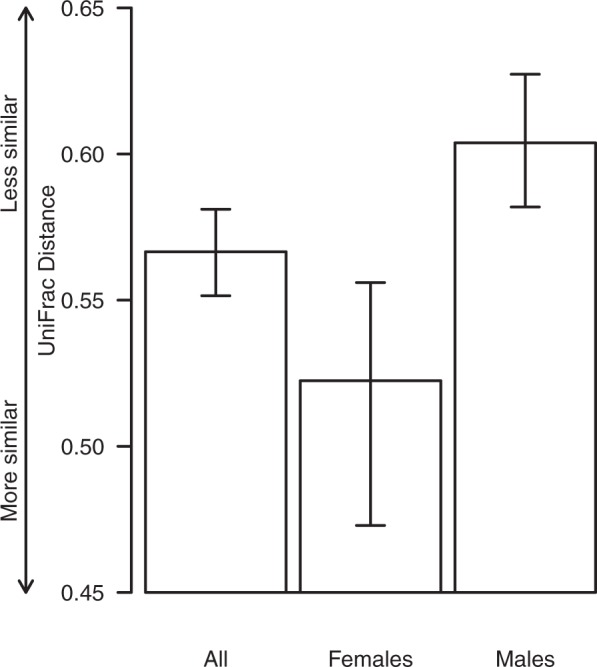
Mean UniFrac distances between pairs of samples that are in extreme quartiles of urinary arsenic for all babies who were formula-fed, for formula-fed males only and for formula-fed females only. Bars represent bootstrapped 95% confidence intervals around the means.

To further investigate sex-specific differences among formula-fed infants, we compared mean UniFrac distances between pairs of samples in extreme quartiles of urinary arsenic among all formula-fed babies and, for male and female formula-fed babies only. In this analysis, we observed that males in extreme quartiles of urinary arsenic concentration had microbiome profiles that were more dissimilar than their female counterparts (Fig. 3).

Among males who were exclusively or partially formula-fed, natural log-transformed urinary arsenic concentration related to the relative abundance of 9 individual OTUs after adjusting for any breastfeeding and urine specific gravity—2 OTUs positively and 7 negatively (Fig. 2 and Supplementary Table S2). Microbes negatively associated with higher urinary arsenic in this subset included *Bifidobacterium*, *Bacteroides*, and *Lactobacillus*, whereas an OTU of the family Lachnospiraceae and another of the genus Streptococcus were positively associated. Associations between natural log-transformed urinary arsenic concentration and the relative abundance of individual OTUs for other stratification groups (formula-fed females, exclusively breastfed males, and exclusively breastfed females) are provided in the Supplementary Information (Supplementary Tables S3–S5).

## Discussion

To our knowledge this is among the first studies of arsenic exposure and the human microbiome. In our analysis of 204 infants from our pregnancy cohort, we identified associations between urinary arsenic in 6-week-old infants and the early intestinal microbiome, both in terms of overall microbiome community composition and among bacterial taxa that are critical for immune training in infancy. We observed that male formula-fed infants may be more susceptible to the effects of arsenic on the microbiota than their female counterparts.

The range of arsenic exposure in well water in our US cohort study in New Hampshire is low in comparison to studies of arsenic exposure in other cohorts such as in Bangladesh^38^. Well water used by the mothers in this cohort had total arsenic concentrations ranging from below the limit of detection to 57.0 *μ*g/L (mean: 1.5 *μ*g/L; median: 0.1 *μ*g/L), with 2% above the EPA maximum contaminant level of 10 *μ*g/L. The infants in our study had urinary arsenic concentrations of ranging from below the limit of detection to 4.8 *μ*g/L (mean: 0.6 *μ*g/L; median: 0.4 *μ*g/L). At these exposure levels, we identified an association with the developing intestinal microbiome, alterations of which associate with major health outcomes^12^.

We found an overall marginal association between infant urinary arsenic and microbiome composition among all infants in the study after adjustment for feeding method and urine specific gravity. Breast milk is known to be low in arsenic in our population and others^15,34,35,51–55^ and exclusively breastfed babies had lower urinary arsenic concentrations than formula or mixed fed infants in our cohort^34^. Previous research by our group showed that this is to the contamination of both formula powder and drinking water used to mix formula^34^, with popular brands of formula containing from 2.2–12.6 ng/g of arsenic, most of it inorganic^56^.

In our study, we identified associations with overall microbiome community composition among formula-fed males but not among breastfed infants or among formula-fed females. Chi and colleagues^50^ reported recently on sex-specific effects of arsenic on the microbiome in mice. In contrast to our finding in humans, female mice exhibited greater susceptibility to microbiome perturbation in association with exposure to arsenic-contaminated drinking water than males^50^. The bacterial communities that colonize mice and humans are distinct, a factor which may explain the different patterns of sex-specific associations between arsenic exposure and microbiome composition; however, taken together, this experimental work in mice and our observational human study suggests that arsenic is one of many factors shaping the gut microbiome of exposed infants and that its effect may be different in males and females. In arsenic-exposed populations in Bangladesh, Mexico, and central Europe, sex has been implicated as a factor influencing the efficiency of inorganic arsenic metabolism^57–59^. In experimental gut simulation models, gut microbes are capable of metabolizing, thiolating and methylating arsenic into more or less toxic forms prior to absorption into the blood stream^4,6,10^. The underlying dynamics of any interactions between arsenic exposure, the gut microbiome and sex are therefore likely complex, as the composition of the gut microbiome may be both a driver and consequence of arsenic exposure, with sex hormones potentially influencing both arsenic metabolism and gut microbiome composition^1,4,6,8–10,50,60^. Metabolomic profiling may offer mechanistic clues.

Arsenic exposure is a risk factor for a range of diseases that are also associated with gut microbial community composition. Numerous epidemiological studies support associations between arsenic exposure and diabetes risk (reviewed in^61^). We found that genera from the phylum Firmicutes and the family Lachnospiraceae were enriched with higher arsenic exposure. In obese mice, intestinal colonization with Lachnospiraceae has been implicated in the development of diabetes^62^. Arsenic has also been shown to have determinantal effects on immune function, even in low doses^63–66^. In our study, Clostridiaceae and the genera *Bacteroides* and *Bifidobacterium*, in particular, were inversely related to arsenic exposure. *Bacteroides* species play a critical role in the maturation of the immune system and help induce regulatory T-cells^67,68^. *Bifidobacterium* also is implicated in immune training in early infancy, and decreases are associated with occurrence of allergy and atopy^69–71^. Studies linking the neonatal gut microbiome composition to development of asthma, identified a depletion of *Bifidobacterium*, *Faecalibacterium*, and *Akkermansia* as being associated with greater risk of asthma at age four^71,72^. Among male infants, negative associations were observed between infant urinary arsenic and *Bifidobacterium*, *Bacteroides* and *Lactobacillus*. The presence of these particular microbes in early life has been implicated in association with complex immune training of the adaptive and innate immune system; absence is associated with decreased immune responsiveness^73^, early onset allergy^27,74^ and asthma and atopy^71^.

The differential abundance of many bacterial taxa along an arsenic exposure gradient that we observed is consistent with the antimicrobial effects of arsenic. For instance, diamino dihydroxy arsenobenzol (arsphenamine) known as Salvarsan was used as an antibiotic to treat syphilis prior to the introduction of penicillin^4,6,10^. Future studies should evaluate a potential role for gut microbiome in a causal pathway linking arsenic with immune function and health outcomes such as respiratory and gastrointestinal disease.

Our study benefited from the careful characterization of arsenic exposure during infancy using advanced methods in arsenic detection and next generation sequencing of the gut microbiome. Nonetheless, findings are limited by the analysis of one time point in infancy at six weeks of life in a single US cohort in New Hampshire. Thus, our results may not be generalizable to other populations, groups with higher levels of arsenic exposure, or at later time points in development. In addition, our observational study design limits our ability to make causal inferences. However, our study draws on one of the largest birth cohorts in the US, with ongoing longitudinal sampling of infants and young children. With ongoing accrual and future collaborations efforts, we hope larger, multi-center studies and prospective analysis of additional time points in infancy can be achieved.

In conclusion, we have identified potential impacts of infant arsenic exposure and differences in overall intestinal microbiome composition and in key taxa that are critical to proper immune training in early life. Our data suggest sex specific differences among formula-fed, arsenic exposed young infants, with male infants experiencing differences in the microbiome with increasing arsenic exposure. Clarifying the impact of low level arsenic exposure on the developing infant microbiome and determining the impact on immune function and health outcomes affords opportunity to alter exposures and impact lifelong health.

## Methods

### The New Hampshire Birth Cohort Study

The New Hampshire Birth Cohort Study (NHBCS) recruited pregnant women ages 18–45 from New Hampshire prenatal clinics beginning at approximately 24 to 28 weeks gestation as previously described^75,76^. Women were eligible if they used a private, unregulated well at the residence occupied since their last menstrual period and were not planning to move. Institutional review board approval was obtained from the Center for the Protection of Human Subjects at Dartmouth, and participants provided written informed consent. All methods were performed in accordance with the relevant guidelines and regulations.

### Infant urine and stool samples

A subset of mothers enrolled in the NHBCS provided infant stool samples collected at the time of regularly scheduled maternal postnatal follow-up visits conducted at approximately 6 weeks post-partum. Infant urine samples were collected using provided diapers (Pampers) containing cotton pads (Shiseido) according to a protocol adapted from Fängström *et al*.^35^. Urine-saturated pads were placed in an acid washed specimen cup and sealed in a polyethylene bag. Stool was collected in provided diapers, sealed in a separate polyethylene bag and frozen in home freezer until transport. Urine and stool samples were transported in a cooler with ice packs and brought to the post-partum visit within 24 hours of collection. Urine samples were maintained at 4 °C and processed within 24 hours. During processing urine was removed from pads, aliquoted into acid washed tubes and frozen at −80 °C. Stool was maintained frozen and thawed at 4 °C prior to processing. Using sterile applicators, 0.5–1 g of stool was aliquoted into cryovial tubes containing 3 ml of RNAlater stabilizer, homogenized by vortexing to create a slurry, maintained at 4 °C for 24 hours and then frozen at −80 °C.

### Urine sample analysis for infant arsenic exposure

Urine samples were analyzed for total arsenic concentration using inductively coupled plasma mass spectrometry at the Dartmouth Trace Metal Analysis Laboratory as described previously^34^. The limit of detection for total urinary arsenic was 0.05 *μ*g/L, and samples with measured arsenic levels below this limit were assigned values of half of the limit of detection, or 0.025 *μ*g/L. Though total urinary arsenic includes arsenobetaine, an organoarsenic compound considered relatively non-toxic, human exposure to arsenobetaine is primarily through the consumption of fish and seafood. In young infants who have not yet been introduced to complementary foods, total arsenic is therefore a reasonable surrogate for exposure to toxic arsenic species.

### Stool sample analysis for infant gut microbiome composition

We performed microbiome characterizations of stool samples as described previously^49^. Briefly, DNA was extracted using the Zymo Research ZR Fecal DNA Mini-prep extraction kit with 0.5 mm bashing beads and sequenced with Illumina tag sequencing of the 16S rRNA gene V4-V5 hypervariable region at the Marine Biological Laboratories in Woods Hole, Massachusetts using established methods^77,78^. Details of sequencing methods, quality control and filtering are described elsewhere^79^.

### Infant feeding

We have previously reported associations between infant feeding method (breast milk vs. formula vs. a combination of breast milk and formula) and infant gut microbiome composition^49^ in the NHBCS. In the present analysis, therefore, we took care to ensure that observed associations between infant arsenic exposure and microbiome composition were not confounded by infant feeding by both adjusting models for infant feeding and by stratification by feeding group. We ascertained infant diet from birth until the time of stool collection by telephone questionnaires that included questions regarding the duration of breastfeeding and the timing of formula introduction, if any. Infants who up until the time of urine and stool collection were fed breastmilk and who had never been given formula were given the dietary status of exclusive breastmilk feeding; infants who had not been breastfed and who had been fed formula only were assigned the status exclusively formula-fed; and infants who had received both breastmilk and formula were identified as having a diet of a combination of breastmilk and formula. Adjusted models included these three dietary categories. Stratified analyses evaluated formula-fed (i.e. exclusively formula-fed and combination fed) subjects as a group and exclusively breastfed subjects as a group. Sample size considerations precluded us from analyzing exclusively formula-fed subjects separately from combination fed subjects; however, our previous research on this cohort indicated that breastfed infants supplemented with formula have microbiome compositions more similar to those fed formula exclusively than to those fed breast milk exclusively^49^.

### Data analysis

We implemented the UCLUST algorithm^80^ in QIIME version 1.9.1^81^ to group sequences of ≥97 percent similarity into open reference operational taxonomic units (OTUs). Bacterial taxonomy was assigned using PyNAST alignment^82^ to the Greengenes reference database^83–85^.

We evaluated the associations between infant urinary arsenic concentration and stool microbiome community composition using generalized UniFrac analysis^86^. To avoid sequencing depth bias, the OTU table was rarefied to the minimum sequencing depth of 10,818 reads in the data set prior to analysis. The phylogenetic tree required for UniFrac analysis was computed using FastTree^87^ and was midpoint rooted. Phylogenetic distances between the microbiome community composition of pairs of infant stool samples were characterized using generalized UniFrac analysis, and relationships between pairwise generalized UniFrac distance matrices and natural log-transformed infant urinary arsenic concentration were evaluated with a permutational multivariate analysis of variance (PERMANOVA) performed with 10,000 permutations. All PERMANOVA models were adjusted for urine specific gravity to control for the effects of urine concentration. While statistical analyses were performed on continuous measures of urinary arsenic concentrations, generalized UniFrac distances were visualized in two-dimensional space using principal coordinates analysis with points colored according to group membership defined by separating subjects at the median urinary arsenic concentration for all subjects of 0.36 *μ*g/L. To further visualize relationships, samples were divided according to infant urinary arsenic concentration quartile, and mean pairwise generalized UniFrac distances were computed for pairs in extreme (first and fourth) quartiles. 95 percent confidence intervals around mean pairwise generalized UniFrac distances were computed using the bias correction with acceleration method.

Associations between infant urinary arsenic concentration and specific OTU relative abundance were modeled by a zero-inflated logistic normal (ZILN) model^88^. We regressed the log-ratio transformations of the OTUs relative abundance on the arsenic exposure adjusting for specific gravity and feeding method. Zero relative abundance values were handled by the zero part of ZILN distribution in the regression model. Estimating-equations approach was employed for the estimation procedure to address the complicated inter-taxa correlations induced by the hierarchical phylogenetic tree structure. The minimax concave penalty^89^ was used to select OTUs and minimize bias in parameter estimation in the regression model. Beta estimates from this model were aggregated based on the lowest taxonomic rank, annotated into a phylogenetic tree and visualized using GraPhlAn v0.9.7 (http://huttenhower.sph.harvard.edu/graphlan)^90^.

## Electronic supplementary material

Supplementary Information

## Data Availability

The datasets generated during and/or analyzed during the current study are available in GENBANK http://www.ncbi.nlm.nih.gov/genbank/ under accession number PRJNA296814 and/or from the corresponding author on reasonable request.
