# Supplementary material for: Sex-specific associations of infants’ gut microbiome with arsenic exposure in a US population
Source: Sci Rep. 2018 Aug 22;8:12627. doi: 10.1038/s41598-018-30581-9 (PMC6105615; doi:10.1038/s41598-018-30581-9)
Supplement: Supplementary file 1 — Supplementary Information [file 41598_2018_30581_MOESM1_ESM.pdf]

SUPPLEMENTARY INFORMATION

Sex-specific associations of infants' gut microbiome with arsenic exposure in a US population

Authors: Anne G. Hoen, PhD[1,2,3,*], Juliette C. Madan, MD, MS[1,2,4], Zhigang Li, PhD[3], Modupe Coker, BDS, PhD[1], Sara N. Lundgren[1], Hilary G. Morrison, PhD[5], Thomas Palys, PhD[2], Brian P. Jackson, PhD[6], Mitchell L. Sogin, PhD[5], Kathryn L. Cottingham, PhD[2,7], Margaret R. Karagas, PhD[1,2]

Affiliations:

[1]Department of Epidemiology, The Geisel School of Medicine at Dartmouth, Hanover, New Hampshire, USA

[2]Children's Environmental Health & Disease Prevention Research Center at Dartmouth, Hanover, New Hampshire, USA

[3]Department of Biomedical Data Science, The Geisel School of Medicine at Dartmouth, Hanover, New Hampshire, USA

[4]Division of Neonatology, Department of Pediatrics, Children's Hospital at Dartmouth, Lebanon, New Hampshire, USA

[5]Josephine Bay Paul Center, Marine Biological Laboratory, Woods Hole, Massachusetts, USA

[6]Department of Earth Sciences, Dartmouth College, Hanover, New Hampshire, USA

[7]Department of Biological Sciences, Dartmouth College, Hanover, New Hampshire, USA

***Corresponding author**: Anne G. Hoen, PhD, Department of Epidemiology, The Geisel School of Medicine at Dartmouth. 1 Medical Center Drive, Lebanon, New Hampshire 03756. Phone: 603-653-6087. Email: anne.g.hoen@dartmouth.edu

Table S1. Results of zero-inflated logistic normal models of taxon-specific asssociations with infant urinary arsenic concentration among N=204 subjects

OTU taxonomic assignment*	Beta	Fold change**
k: Bacteria; p: Actinobacteria; c: Actinobacteria; o: Bifidobacteriales; f: Bifidobacteriaceae; g: *Bifidobacterium*; s: unassigned	-0.89	0.54
k: Bacteria; p: Bacteroidetes; c: Bacteroidia; o: Bacteroidales; f: Bacteroidaceae; g: *Bacteroides*; s: unassigned	-1.26	0.42
k: Bacteria; p: Bacteroidetes; c: Bacteroidia; o: Bacteroidales; f: Bacteroidaceae; g: *Bacteroides*; s: unassigned	-1.20	0.43
k: Bacteria; p: Bacteroidetes; c: Bacteroidia; o: Bacteroidales; f: Porphyromonadaceae; g: *Parabacteroides*; s: unassigned	-0.87	0.55
k: Bacteria; p: Bacteroidetes; c: Bacteroidia; o: Bacteroidales; f: Rikenellaceae; g: unassigned; s: unassigned	-1.21	0.43
k: Bacteria; p: Bacteroidetes; c: Bacteroidia; o: Bacteroidales; f: Rikenellaceae; g: unassigned; s: unassigned	-0.24	0.84
k: Bacteria; p: Firmicutes; c: Bacilli; o: Lactobacillales; f: Enterococcaceae; g: *Enterococcus*; s: unassigned	0.19	1.14
k: Bacteria; p: Firmicutes; c: Bacilli; o: Lactobacillales; f: Lactobacillaceae; g: *Lactobacillus*; s: unassigned	-0.04	0.97
k: Bacteria; p: Firmicutes; c: Bacilli; o: Lactobacillales; f: Lactobacillaceae; g: *Lactobacillus*; s: unassigned	1.26	2.39
k: Bacteria; p: Firmicutes; c: Bacilli; o: Lactobacillales; f: Streptococcaceae; g: *Streptococcus*; s: unassigned	0.13	1.09
k: Bacteria; p: Firmicutes; c: Clostridia; o: Clostridiales; f: Eubacteriaceae; g: *Pseudoramibacter Eubacterium*; s: unassigned	-0.84	0.56
k: Bacteria; p: Firmicutes; c: Clostridia; o: Clostridiales; f: Lachnospiraceae; g: unassigned; s: unassigned	-1.15	0.45
k: Bacteria; p: Firmicutes; c: Clostridia; o: Clostridiales; f: Lachnospiraceae; g: unassigned; s: unassigned	-0.34	0.79
k: Bacteria; p: Firmicutes; c: Clostridia; o: Clostridiales; f: Lachnospiraceae; g: unassigned; s: unassigned	-0.12	0.92
k: Bacteria; p: Firmicutes; c: Clostridia; o: Clostridiales; f: Lachnospiraceae; g: *Blautia*; s: unassigned	2.13	4.38
k: Bacteria; p: Firmicutes; c: Clostridia; o: Clostridiales; f: Lachnospiraceae; g: *Coprococcus*; s: unassigned	-0.57	0.67
k: Bacteria; p: Firmicutes; c: Clostridia; o: Clostridiales; f: Lachnospiraceae; g: *Dorea*; s: unassigned	-0.16	0.89
k: Bacteria; p: Firmicutes; c: Clostridia; o: Clostridiales; f: Lachnospiraceae; g: *Dorea*; s: unassigned	0.39	1.31
k: Bacteria; p: Firmicutes; c: Clostridia; o: Clostridiales; f: Ruminococcaceae; g: *Ruminococcus*; s: unassigned	5.68	51.44
k: Bacteria; p: Proteobacteria; c: Gammaproteobacteria; o: Enterobacteriales; f: Enterobacteriaceae; g: unassigned; s: unassigned	-1.86	0.28
k: Bacteria; p: Proteobacteria; c: Gammaproteobacteria; o: Xanthomonadales; f: Xanthomonadaceae; g: unassigned; s: unassigned	1.36	2.57
k: Bacteria; p: Proteobacteria; c: Gammaproteobacteria; o: Xanthomonadales; f: Xanthomonadaceae; g: unassigned; s: unassigned	0.75	1.68

*OTU: operational taxonomic unit; k: kingdom; p: phylum; c: class; o:order; f: family; g: genus; s: species

**Fold increase in relative abundance for every fold increase in natural log transformed infant urinary arsenic concentration

Table S2. Results of zero-inflated logistic normal models of taxon-specific asssociations with infant urinary arsenic concentration among N=33 formula-fed male subjects

OTU taxonomic assignment*	Beta	Fold change**
k: Bacteria; p: Actinobacteria; c: Actinobacteria; o: Actinomycetales; f: Actinomycetaceae; g: *Actinomyces*; s: unassigned	-0.84	0.56
k: Bacteria; p: Actinobacteria; c: Actinobacteria; o: Bifidobacteriales; f: Bifidobacteriaceae; g: *Bifidobacterium*; s: unassigned	-1.42	0.37
k: Bacteria; p: Actinobacteria; c: Actinobacteria; o: Bifidobacteriales; f: Bifidobacteriaceae; g: *Bifidobacterium*; s: unassigned	-0.02	0.99
k: Bacteria; p: Bacteroidetes; c: Bacteroidia; o: Bacteroidales; f: Bacteroidaceae; g: *Bacteroides*; s: *fragilis*	-0.55	0.68
k: Bacteria; p: Bacteroidetes; c: Bacteroidia; o: Bacteroidales; f: Bacteroidaceae; g: *Bacteroides*; s: *ovatus*	-0.66	0.63
k: Bacteria; p: Firmicutes; c: Bacilli; o: Lactobacillales; f: Lactobacillaceae; g: *Lactobacillus*; s: unassigned	-1.50	0.35
k: Bacteria; p: Firmicutes; c: Bacilli; o: Lactobacillales; f: Lactobacillaceae; g: *Lactobacillus*; s: *zeae*	-0.78	0.58
k: Bacteria; p: Firmicutes; c: Bacilli; o: Lactobacillales; f: Streptococcaceae; g: *Streptococcus*; s: unassigned	0.91	1.88
k: Bacteria; p: Firmicutes; c: Clostridia; o: Clostridiales; f: Lachnospiraceae; g: unassigned; s: unassigned	0.20	1.15

*OTU: operational taxonomic unit; k: kingdom; p: phylum; c: class; o:order; f: family; g: genus; s: species

**Fold increase in relative abundance for every fold increase in natural log transformed infant urinary arsenic concentration

Table S3. Results of zero-inflated logistic normal models of taxon-specific asssociations with infant urinary arsenic concentration among N=23 formula-fed female subjects

OTU taxonomic assignment*	Beta	Fold change**
k: Bacteria; p: Actinobacteria; c: Actinobacteria; o: Bifidobacteriales; f: Bifidobacteriaceae; g: *Bifidobacterium*; s: unassigned	-0.12	0.92
k: Bacteria; p: Firmicutes; c: Bacilli; o: Lactobacillales; f: Lactobacillaceae; g: *Lactobacillus*; s: unassigned	0.64	1.56
k: Bacteria; p: Firmicutes; c: Bacilli; o: Lactobacillales; f: Streptococcaceae; g: *Streptococcus*; s: unassigned	-0.06	0.96
k: Bacteria; p: Firmicutes; c: Clostridia; o: Clostridiales; f: Lachnospiraceae; g: *Blautia*; s: unassigned	1.26	2.40
k: Bacteria; p: Firmicutes; c: Clostridia; o: Clostridiales; f: Lachnospiraceae; g: unassigned; s: unassigned	3.27	9.66

*OTU: operational taxonomic unit; k: kingdom; p: phylum; c: class; o:order; f: family; g: genus; s: species

**Fold increase in relative abundance for every fold increase in natural log transformed infant urinary arsenic concentration

Table S4. Results of zero-inflated logistic normal models of taxon-specific asssociations with infant urinary arsenic concentration among N=85 exclusively breastfed male subjects

OTU taxonomic assignment*	Beta	Fold change**
k: Bacteria; p: Bacteroidetes; c: Bacteroidia; o: Bacteroidales; f: Bacteroidaceae; g: *Bacteroides* ; s: *caccae*	-0.61	0.66
k: Bacteria; p: Bacteroidetes; c: Bacteroidia; o: Bacteroidales; f: Bacteroidaceae; g: *Bacteroides* ; s: unassigned	-0.31	0.81
k: Bacteria; p: Bacteroidetes; c: Bacteroidia; o: Bacteroidales; f: Bacteroidaceae; g: *Bacteroides* ; s: unassigned	-1.25	0.42
k: Bacteria; p: Bacteroidetes; c: Bacteroidia; o: Bacteroidales; f: Bacteroidaceae; g: *Bacteroides* ; s: unassigned	-0.05	0.97
k: Bacteria; p: Bacteroidetes; c: Bacteroidia; o: Bacteroidales; f: Bacteroidaceae; g: Bacteroides; s: unassigned	-0.42	0.75
k: Bacteria; p: Bacteroidetes; c: Bacteroidia; o: Bacteroidales; f: Bacteroidaceae; g: *Bacteroides* ; s: unassigned	-0.01	0.99
k: Bacteria; p: Bacteroidetes; c: Bacteroidia; o: Bacteroidales; f: Bacteroidaceae; g: *Bacteroides* ; s: unassigned	-0.28	0.82
k: Bacteria; p: Bacteroidetes; c: Bacteroidia; o: Bacteroidales; f: Bacteroidaceae; g: *Bacteroides* ; s: unassigned	-0.32	0.80
k: Bacteria; p: Bacteroidetes; c: Bacteroidia; o: Bacteroidales; f: Bacteroidaceae; g: *Bacteroides* ; s: unassigned	-0.89	0.54
k: Bacteria; p: Firmicutes; c: Bacilli; o: Lactobacillales; f: Streptococcaceae; g: *Streptococcus* ; s: *anginosus*	-0.44	0.74
k: Bacteria; p: Firmicutes; c: Clostridia; o: Clostridiales; f: Eubacteriaceae; g: *Pseudoramibacter Eubacterium* ; s: unassigned	-0.63	0.65
k: Bacteria; p: Firmicutes; c: Clostridia; o: Clostridiales; f: Lachnospiraceae; g: unassigned; s: unassigned	-1.36	0.39
k: Bacteria; p: Firmicutes; c: Clostridia; o: Clostridiales; f: Lachnospiraceae; g: unassigned; s: unassigned	-0.66	0.63
k: Bacteria; p: Firmicutes; c: Clostridia; o: Clostridiales; f: Ruminococcaceae; g: unassigned; s: unassigned	9.94	984.89
k: Bacteria; p: Proteobacteria; c: Gammaproteobacteria; o: Enterobacteriales; f: Enterobacteriaceae; g: unassigned; s: unassigned	-3.67	0.08
k: Bacteria; p: Proteobacteria; c: Gammaproteobacteria; o: Xanthomonadales; f: Xanthomonadaceae; g: unassigned; s: unassigned	0.86	1.81

*OTU: operational taxonomic unit; k: kingdom; p: phylum; c: class; o:order; f: family; g: genus; s: species

**Fold increase in relative abundance for every fold increase in natural log transformed infant urinary arsenic concentration

Table S5. Results of zero-inflated logistic normal models of taxon-specific asssociations with infant urinary arsenic concentration among N=58 exclusively breastfed female subjects

OTU taxonomic assignment*	Beta	Fold change**
k: Bacteria; p: Bacteroidetes; c: Bacteroidia; o: Bacteroidales; f: Bacteroidaceae; g: *Bacteroides* ; s: *fragilis*	0.14	1.11
k: Bacteria; p: Firmicutes; c: Clostridia; o: Clostridiales; f: Lachnospiraceae; g: unassigned; s: unassigned	-0.3	0.81
k: Bacteria; p: Firmicutes; c: Clostridia; o: Clostridiales; f: Lachnospiraceae; g: unassigned; s: unassigned	-0.3	0.82
k: Bacteria; p: Firmicutes; c: Clostridia; o: Clostridiales; f: Veillonellaceae; g: *Veillonella; s: unassigned*	-1.2	0.44
k: Bacteria; p: Firmicutes; c: Bacilli; o: Lactobacillales; f: Lactobacillaceae; g: *Lactobacillus* ; s: unassigned	-0.2	0.87
k: Bacteria; p: Firmicutes; c: Clostridia; o: Clostridiales; f: Veillonellaceae; g: *Phascolarctobacterium* ; s: unassigned	-0.9	0.53
k: Bacteria; p: Bacteroidetes; c: Bacteroidia; o: Bacteroidales; f: Rikenellaceae; g: unassigned; s: assigned	-1	0.48
k: Bacteria; p: Bacteroidetes; c: Bacteroidia; o: Bacteroidales; f: Porphyromonadaceae; g: *Parabacteroides* ; s: *distasonis*	-1	0.49
k: Bacteria; p: Bacteroidetes; c: Bacteroidia; o: Bacteroidales; f: Bacteroidaceae; g: *Bacteroides* ; s: *ovatus*	-0.6	0.65
k: Bacteria; p: Bacteroidetes; c: Bacteroidia; o: Bacteroidales; f: Porphyromonadaceae; g: *Parabacteroides* ; s: *distasonis*	-0.1	0.91
k: Bacteria; p: Actinobacteria; c: Actinobacteria; o: Bifidobacteriales; f: Bifidobacteriaceae; g: *Bifidobacterium* ; s: unassigned	-0	0.99
k: Bacteria; p: Actinobacteria; c: Actinobacteria; o: Bifidobacteriales; f: Bifidobacteriaceae; g: *Bifidobacterium* ; s: *adolescentis*	-0.1	0.95
k: Bacteria; p: Bacteroidetes; c: Bacteroidia; o: Bacteroidales; f: Bacteroidaceae; g: *Bacteroides* ; s: unassigned	-2	0.25
k: Bacteria; p: Firmicutes; c: Clostridia; o: Clostridiales; f: Lachnospiraceae; g: unassigned ; s: unassigned	-0.4	0.76
k: Bacteria; p: Bacteroidetes; c: Bacteroidia; o: Bacteroidales; f: Bacteroidaceae; g: *Bacteroides* ; s: unassigned	-1	0.50
k: Bacteria; p: Bacteroidetes; c: Bacteroidia; o: Bacteroidales; f: Bacteroidaceae; g: *Bacteroides* ; s: unassigned	-0.4	0.73
k: Bacteria; p: Firmicutes; c: Erysipelotrichi; o: Erysipelotrichales; f: Erysipelotrichaceae; g: *Eubacterium* ; s: *dolichum*	-0.8	0.56
k: Bacteria; p: Bacteroidetes; c: Bacteroidia; o: Bacteroidales; f: Porphyromonadaceae; g: *Parabacteroides* ; s: unassigned	-0.4	0.77

*OTU: operational taxonomic unit; k: kingdom; p: phylum; c: class; o:order; f: family; g: genus; s: species

**Fold increase in relative abundance for every fold increase in natural log transformed infant urinary arsenic concentration

Supplementary figure legend

Fig. S1. Principal coordinate plot of generalized UniFrac distance matrix comparing microbiome community profiles according to urinary arsenic concentration. (A) N=23 formula-fed females; (B) N=85 exclusively breastfed males; (C) N=58 exclusively breastfed females. Corresponding plots for other groups (all study subjects together and N=33 formula-fed males) are presented in the main manuscript body (Fig. 1). Statistical testing was performed using a continuous measure of arsenic exposure, the natural log of urinary arsenic concentration; however, for visualization purposes, here subjects were divided at the median urinary arsenic concentration of 0.36 μg/L.

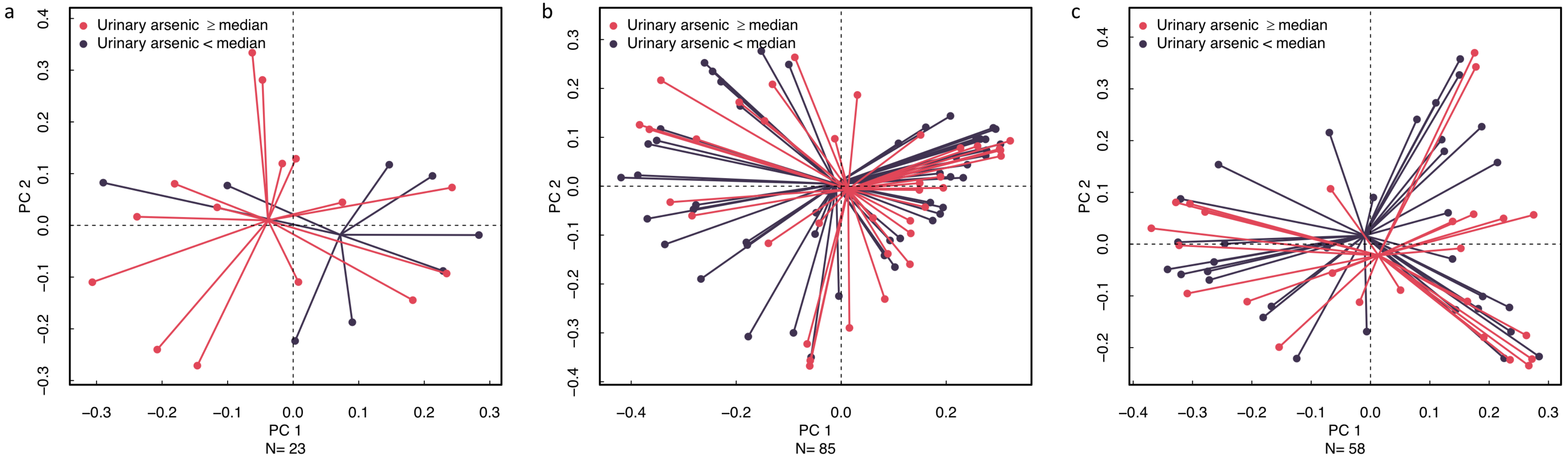
a
b
c
Urinary arsenic ≥ median
Urinary arsenic < median
PC 1
PC 2
N= 23
N= 85
N= 58
−0.3
−0.2
−0.1
0.0
0.1
0.2
0.3
0.4
−0.4
